# Monitoring Physiological Changes in Haloarchaeal Cell during Virus Release

**DOI:** 10.3390/v8030059

**Published:** 2016-02-24

**Authors:** Julija Svirskaitė, Hanna M. Oksanen, Rimantas Daugelavičius, Dennis H. Bamford

**Affiliations:** 1 Department of Biosciences, Institute of Biotechnology, University of Helsinki, Viikinkaari 9, 00014 Helsinki, Finland; julija.svirskaite@helsinki.fi (J.S.); hanna.oksanen@helsinki.fi (H.M.O.); 2 Department of Biochemistry, Vytautas Magnus University, Vileikos g. 8, 44404 Kaunas, Lithuania; r.daugelavicius@gmf.vdu.lt

**Keywords:** *Haloarcula hispanica*, icosahedral membrane-containing virus SH1, icosahedral tailed virus HHTV-1, spindle-shaped virus His1, pleomorphic virus His2, potentiometry, virus life cycle, *Pleolipoviridae*, *Sphaerolipoviridae*

## Abstract

The slow rate of adsorption and non-synchronous release of some archaeal viruses have hindered more thorough analyses of the mechanisms of archaeal virus release. To address this deficit, we utilized four viruses that infect *Haloarcula hispanica* that represent the four virion morphotypes currently known for halophilic euryarchaeal viruses: (1) icosahedral internal membrane-containing SH1; (2) icosahedral tailed HHTV-1; (3) spindle-shaped His1; and (4) pleomorphic His2. To discern the events occurring as the progeny viruses exit, we monitored culture turbidity, as well as viable cell and progeny virus counts of infected and uninfected cultures. In addition to these traditional metrics, we measured three parameters associated with membrane integrity: the binding of the lipophilic anion phenyldicarbaundecaborane, oxygen consumption, and both intra- and extra-cellular ATP levels.

## 1. Introduction

Archaea are widespread in diverse ecosystems and dominate environments with elevated temperature, alkalinity, acidity, or salinity [1]. As for other unicellular organisms that thrive in harsh conditions, their cell envelope provides a protective barrier and also has a critical role in maintaining selective membrane permeability. However, archaeal membrane composition differs fundamentally from that of bacteria and eukaryotes. In archaea, isoprenoid hydrocarbon side chains are linked via an ether bond to the phosphate backbone [2], whereas, in bacteria and eukaryotes, fatty acids are linked by an ester bond. In general, archaea have only a single membrane that is typically surrounded by a paracrystalline protein layer, the S-layer. This outer protein layer gives the archaeal envelope its rigid surface and bestows on the cell a particular shape and surface qualities [3]. This, combined with the cytoplasmic membrane, forms a challenging barrier for archaeal viruses during both infection and progeny egress.

Since discovery of archaeal viruses in 1974 by Torsvik and Dundas [4], more than 130 archaeal viruses have been described, the majority of which (~90) infect halophilic euryarchaea [5,6]. The most diverse and unique archaeal virus morphologies have been found among viruses infecting thermophilic crenarchaea [7], whereas only four morphotypes have been identified among the euryarchaeal viruses: icosahedral tailed, spindle-shaped, pleomorphic, and icosahedral with an internal membrane [5].

The mechanisms employed by archaeal viruses to release their progeny from the host cell have been rather elusive [8]. Two crenarchaeal viruses, *Sulfolobus* turreted icosahedral virus (STIV) and *Sulfolobus islandicus* rod-shaped virus 2 (SIRV2), are unique so far in utilizing an exit mechanism that involves the formation of pyramid structures in the cell membrane that open to release the progeny virions [9,10]. Among the euryarchaeal viruses, the only known gene product that could assist in virus release is a lytic enzyme, pseudomurein endoisopeptidase, identified in methanogenic euryarchaeal viruses ψM1 and ψM2 and prophage ψM100 [11,12]. However, the function of the protein product has not been demonstrated, and no gene product active against archaeal S-layer has been identified yet. In contrast, several exit mechanisms have been described for DNA bacteriophages (phages). Numerous double-stranded (ds) DNA phages use a holin-endolysin system [13,14], whereas those with a small, single-stranded (ss) DNA genomes utilize a single-gene lysis mechanism [15] and filamentous phages are continuously extruded from their hosts [16]. Thus far, no archaeal virus genes display any sequence similarity to phage genes encoding exit-related functions. However, although no putative holins have been identified in the genomes of archaeal viruses, some have been found in archaeal genomes [13]. Probably, the fundamental difference in the cell envelopes of bacteria and archaea is one of the main factors driving the evolution of the divergent egress strategies of archaeal viruses and phages [17].

The diverse halophilic euryarchaeal viruses provide an opportunity to investigate these questions. Currently, only four haloarchaeal viruses have been shown to be temperate (φCh1, φH, SNJ1, and SNJ2), each either residing as a plasmid or integrating into the host cell chromosome [18,19,20,21,22]. The known haloarchaeal virus release mechanisms can be divided into two major categories: (i) those where the cell membrane is disrupted by host cell lysis; and (ii) those where the membrane remains intact (e.g., release by budding). Numerous halophilic archaeal viruses release their progeny continuously, apparently without causing host cell lysis [23,24]. A useful system for further investigation is provided by the halophilic euryarchaeon *Haloarcula hispanica* [25] that is infected by viruses representing the four known halophilic archaeal virus morphotypes [26,27,28,29] and also carries proviruses in its chromosome [19]. Four viruses were selected for this study, one from each morphotype: the icosahedral, internal membrane-containing virus SH1, spindle-shaped virus His1, pleomorphic virus His2, and icosahedral *Haloarcula hispanica* tailed virus 1 (HHTV-1) (Table 1). The genomes of all four are linear dsDNA and range in size between 15 and 50 kbp. When cultured in *Har. hispanica* cells, all four viruses reach high titers in the culture medium (Table 1). Receptors have not yet been identified for any euryarchaeal viruses, including these four.

Among the icosahedral viruses with an internal membrane, to date five (SH1, HHIV-2, PH1, SNJ1, and HCIV-1) are known to infect halophilic euryarchaeal hosts, either *Haloarcula* or *Natrinema* strains [18,30,31,32,33]. Only SNJ1 is known to be temperate, switching between lytic and lysogenic replication modes in a salt-concentration dependent manner [18,34]. SH1, the best-studied one and the one included in this study, recognizes host cells via horn-like spike complexes located at the five-fold vertices [31,35,36]. Host cell binding is most effective in high salinity [29]. Structurally SH1 is very similar to HHIV-2 that also infects *Har. hispanica*, but differs in that the host recognition spikes of HHIV-2 are pentameric, propeller-like complexes [35,37]. The lipids of SH1 are selectively acquired from the host plasma membrane during assembly. Progeny viruses are released from the host cell 5–6 h post infection (p.i.), with an average burst size of about 200 [27,31]. Thin-section transmission electron microscopy (TEM) analysis of SH1 infected *Har. hispanica* cells suggests a release mechanism based on cell membrane disruption [31]. In addition to *Har. hispanica*, SH1 also replicates in a *Halorubrum* isolate that resembles *Halorubrum sodomense* [31].

The icosahedral tailed virus HHTV-1 morphologically resembles phages of the *Siphoviridae* family (order *Caudovirales*) [29] and is the least studied of the *Har. hispanica* viruses included here (Table 1). Its genome architecture is mosaic, like those of the siphoviruses, a group whose genomes also display a high degree of sequence diversity [38,39,40]. Its life cycle is unexplored, but it adsorbs slowly to *Har. hispanica* [29]. As typical for halophilic siphoviruses, HHTV-1 has a very narrow host range; it is known to infect only its isolation host *Har. hispanica* and its close relative *Haloarcula* sp. PV7 [41].

The only described euryarchaeal spindle-shaped virus, His1, resembles the short-tailed, spindle-shaped fuselloviruses that infect crenarchaea [24,26,42]. The flexible His1 virion is composed of only one major capsid protein (MCP) species; that MCP is lipid-modified, but the virion lacks a lipid bilayer [24,43]. The spindle-shaped virion transforms into a tube that most probably facilitates DNA delivery during infection [43,44]. His1 infection leads to constant virus release without cell lysis and with no retardation of cell growth in culture until the late logarithmic stage [24,28]. Thin-section TEM of infected cells has not yet captured the exiting of any progeny His1 virions [24].

Although His2 virus had been previously suggested to be spindle-shaped [28], it was later found to be one of the pleomorphic archaeal viruses. These viruses have a very simple virion architecture. The chromosome is surrounded by a membrane vesicle containing but a few major protein species [23]. This unique group was recently proposed to form a new viral family, the “Pleolipoviridae” [45]. During virion assembly His2 acquires its lipids non-selectively from the host cell membrane, probably as the virions exit the cell by budding. Virion production slows host growth [23]. His2, like most of the other archaeal pleomorphic viruses, is specific to a single host [23,28].

Typically insights into virion exit strategies can be gained by one-step growth experiments that include measurement of culture turbidity and counts of progeny viruses and viable cells, supplemented with thin section TEM. However, the slow and sometimes inefficient binding of halophilic viruses to their cellular receptor molecules, long host cell generation times, and non-synchronous virus egress make the determination of archaeal virus exit strategies challenging. The literature holds contradictory proposals concerning the release mechanisms used by some halophilic archaeal viruses [28,31,46,47] showing that the traditional metrics are not always sufficient to warrant the conclusions drawn. Here, we investigated the mechanisms employed by four diverse haloarchaeal viruses by monitoring not only host culture turbidity and progeny virus production, but also changes in the euryarchaeal cell envelope during virus exit. The latter was assessed by measuring the concentration of phenyldicarbaundecaborane anion (PCB^−^) and the level of dissolved oxygen in the medium [48]. These data were accompanied by tracking intra- and extracellular ATP changes [48]. While this potentiometric approach has been previously used to study the life cycles of several phages in moderate environments [48,49,50,51,52,53], here we demonstrate that these techniques can also be applied to viruses thriving in extreme salinities (>3 M NaCl).

## 2. Materials and Methods

### 2.1. Cells, Viruses and Growth of Viruses

In this study, we investigated *Har. hispanica* [25] and four of its viruses: SH1 [31], HHTV-1 [29], His1, and His2 [26] (Table 1). Cultures were grown aerobically at 37 °C in modified growth medium (MGM) containing artificial salt water (SW) (for details, see [54,55]). Broth, solid, and soft-agar media contained 23%, 20%, and 18% SW, respectively. For virus production, suitable virus dilutions were used to obtain semi-confluent plates. To avoid drying during culture, a cup of water was placed with the plates in closed plastic boxes. The soft agar layer of the semiconfluent plates was collected, mixed with 2 mL of MGM per plate, and the resulting suspension was incubated with shaking for 2 h at 37 °C. Soft agar and cell debris were removed by centrifugation (Sorvall SA600 rotor, 9500 rpm, 20 min, +4 °C). The supernatant (virus stock) was stored at +4 °C. Virus titers (pfu/mL) were determined by plaque assay [56]. Culture turbidity was monitored (A_550_), and viable cell counts (cfu/mL) were measured when appropriate. Cells were disrupted by incubation in boiling water for 5 min.

### 2.2. Adsorption Assay

The adsorption rate constant of His2 was determined by infecting *Har. hispanica* culture (~1 × 10^9^ cfu/mL) with His2 virus stock using a multiplicity of infection (MOI) of ~1 × 10^−4^. MGM medium without cells was used as a control. The mixtures were incubated at +37 °C with aeration. Samples taken at different time points were diluted 1:100 in MGM broth. The cells were removed by centrifugation (Biofuge table centrifuge, 13,000 rpm, 5 min, +20 °C) and the number of free viruses in the supernatant was determined by plaque assay. The adsorption rate constant (*k*) was calculated using the formula *k* = 2.3 × log (*P*_0_/*P*) / (*B*)*t*, where *B* is the cell concentration, *P*_0_ is the concentration of free viruses at the beginning, *P* is the concentration of free viruses at the end, and *t* is the period of time [56].

### 2.3. On-Line Electrochemical Measurements during Virus Infection

*Har. hispanica* cells grown in flask culture to 5 × 10^8^ cfu/mL (A_550_ of 1) were infected using the virus stock to give an MOI of 20. For the uninfected control, the same volume of MGM medium was substituted for the virus stock. Cultures were incubated for 2 h, and then the unadsorbed viruses were removed by collecting the cells by centrifugation (Sorvall SA600 rotor, 5000 rpm, 10 min, +20 °C). The cells were resuspended in preheated MGM (+37 °C) to yield a six-fold concentrated cell suspension and transferred immediately into the 50 mL reaction vessels for measurements. Growth of the cells continued in the reaction vessels (37 °C) covered with aluminum foil to prevent evaporation. Turbidity (A_550_) was always followed during growth and electrochemical measurements in the vessels. Number of progeny viruses was determined by plaque assay starting 4 h p.i. To determine the concentration of phenyldicarbaundecaborane (PCB^−^) in the medium, the amount of dissolved oxygen and the ATP content (see Section 2.4), the uninfected and infected cells were grown in the vessels. For PCB^−^ measurements, before adding the cells the electrode was calibrated by adding PCB^−^ to the medium to the final concentration of 3 µM.

The concentration of PCB^−^ was determined using selective electrodes either prepared as previously described [57,58] or as defined below. To synthetize PCB^−^ sensitive membranes, we tested different plasticizers (2-nitrophenyl octyl ether, NPOE (Sigma, St. Louis, MO, USA); dioctyl phthalate (Fluka, Buchs, Switzerland); dibutyl phthalate (Sigma) [59]), concentrations of poly-vinyl chloride (PVC; 6%, 10% (*w*/*v*)), and concentrations of PCB^−^ (0.25 mM and 0.1 mM). In addition, membrane thickness as well as electrode regeneration and storage conditions were optimized. Electrodes were prepared at room temperature. The final solution for membrane synthesis consisted of diluent tetrahydrofuran (THF; Fluka), 6% (*w*/*v*) PVC (Aldrich, St. Louis, MO, USA), 270 mM plasticizer NPOE, and the ion pair of PCB^−^ (0.05 mM) with tetraphenylphosphonium (0.05 mM TPP^+^; Sigma). The potassium salt of PCB^−^ was provided by Aldona Beganskiene, Department of Inorganic Chemistry, Vilnius University. A tip of the glass tube (inner diameter of 0.6 cm and length of 5.5 cm, Laborexin, Helsinki, Finland) was immersed into the membrane synthesis solution to obtain a thin film, which was dried at room temperature. The immersion was repeated once. After drying of the membrane, the final thickness of the coating was obtained by casting three thin layers of the membrane synthesizing solution (45 µL each) on the film (inside the tube). Before each measurement the membrane was soaked in 1 mM PCB^−^ (in distilled water) for 12 h and then in 1 μM PCB^−^ for 24 h. For longer periods of storage, the membranes were dried and stored at room temperature. The glass tube with a membrane was filled with a solution of 1 mM PCB^−^ in 150 mM NaCl, and then attached to the Ag-AgCl half-cell electrode. The Ag-AgCl reference electrode (Thermo, St. Louis, MO, USA, Orion S/junction) was indirectly connected to the reaction vessel through an agar salt bridge.

A selective electrode (Thermo, Orion 97-08 electrode) was used to determine on-line the amount of dissolved oxygen in the medium [48]. Solid Na_2_S_2_O_5_ was added to deplete oxygen to obtain the zero base line (0%). The maximum dissolved oxygen (100%) was registered before adding the cells to the aerated medium.

For data collection, the electrodes were connected to potential amplifying system with an ultralow input bias current operational amplifier AD549JH (Analog Devices, Norwood, MA, USA). The signal amplifier was connected to a computer through data acquisition system PowerLab 8/35 (ADInstruments, Oxford, UK).

### 2.4. ATP Measurements during Virus Infection

To measure intra- and extracellular ATP content, 110 µL samples were taken from the reaction vessels (see above) and the cells collected by centrifugation (Biofuge table centrifuge, 5 min, 13,000 rpm, +25 °C). The supernatant was kept and the pellets were resuspended into the original volume of MGM. Twenty-five microliter samples were withdrawn from the 110 µL samples of both the supernatant and the pellet fractions and the ATP contents were measured using the ATP Biomass Kit (BioThema, Handen, Sweden) and the Turner BioSystems 20/20 luminometer (Promega, Sunnyvale, CA, USA) with two injectors. During all measurements the volumes of the sample, Extractant (BioThema) and luciferin/luciferase reagent (ATP Reagent; BioThema) were 25 µL, 25 µL, and 100 µL, respectively. To measure the amount of ATP the extractant and ATP reagent were injected three seconds after the sample was taken. The light emissions of the extracellular and intracellular ATP were measured after 10 and 150 s (to ensure that cell envelope was destroyed), respectively. For calibration, 25 µL of ATP standard (100 nM) was measured as the extracellular ATP.

## 3. Results

### 3.1. Adsorption Rate Constant of Pleomorphic Virus His2 to *Har. hispanica*

**Figure 1:**
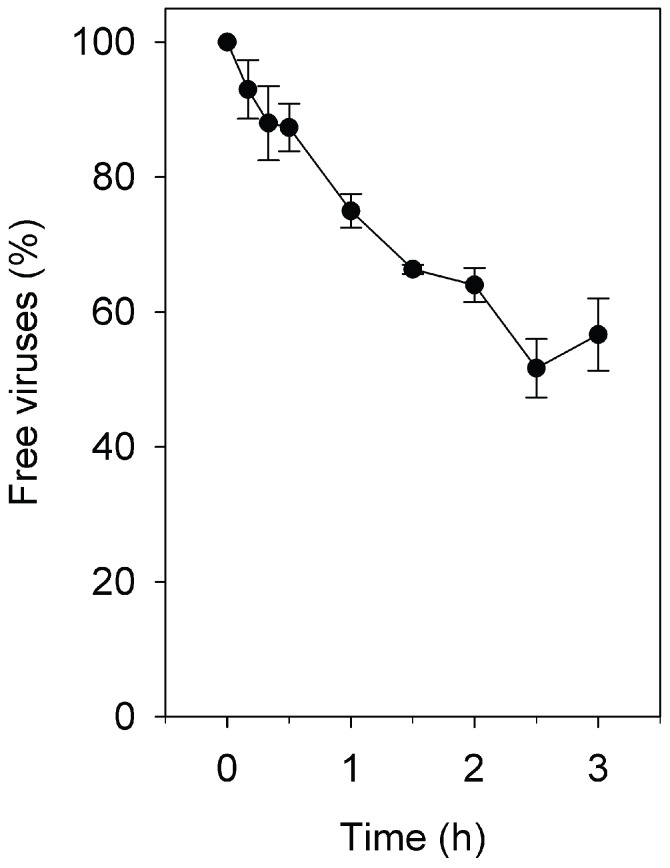
Adsorption efficiency of His2 to *Haloarcula hispanica* cells at 37 °C with aeration. The number of unadsorbed viruses was determined by plaque assay. Linear regression was calculated for 0–150 min (*r*^2^ = 0.97). The last point (180 min) shows the effect of release of the first progeny viruses (see also Figure 3D).

The adsorption rate constants for SH1, HHTV-1, and His1 have been previously determined (Table 1) [24,29,30]. The adsorption rate of His2 was determined here by mixing viruses and host cells at low MOI and determining the reduction in the unadsorbed virus fraction by plaque assay. No reduction in the plaque number in the control (without the cells) was detected. At 2.5 h p.i., ~50% of the viruses were adsorbed (Figure 1). The slight increase in the number of unadsorbed viruses 3 h p.i. was due to the release of progeny viruses. The adsorption rate constant calculated for His2 based on the initial 2 h adsorption period was 5.0 × 10^−12^ mL/min (Table 1).

### 3.2. Monitoring the Turbidity and Counts of Viable Cells and Progeny Viruses during Virus Production

**Figure 2:**
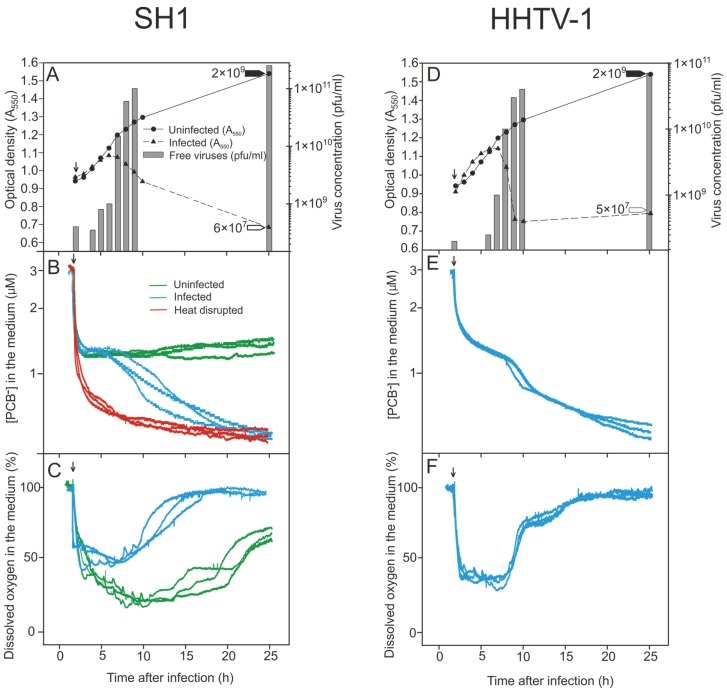
Growth parameters and physiological changes in *Haloarcula hispanica* during infection by (**A**–**C**) SH1 and (**D**,**F**) HHTV-1 (MOI of 20). Unadsorbed viruses were removed at 2 h p.i. and measurements were begun at 2 h 15 min p.i. (indicated by arrows) and were carried out in MGM medium at 37 °C with aeration. (**A**,**D**) Turbidities of the infected and uninfected cultures; the number of free progeny viruses (pfu/mL) in the infected cultures; and the number of viable cells (cfu/mL) at 25 h p.i. in the uninfected (**black** arrow head) and infected cultures (**white** arrow head); (**B**,**E**) PCB^−^ binding in the presence of PCB^−^ (calibrated with 3 µM PCB^−^) to infected, uninfected, and heat disrupted (showing the maximal binding) *Har. hispanica* cells (*n* = 3); (**C**,**F**) The level of dissolved oxygen in the medium of infected and uninfected *Har. hispanica* cells (*n* = 3).

**Figure 3:**
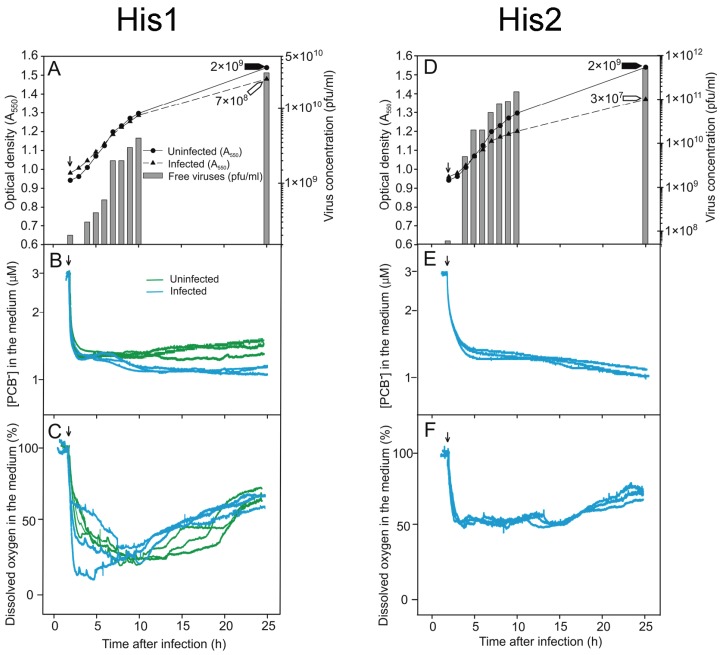
Growth parameters and physiological changes in *Haloarcula hispanica* during (**A**–**C**) His1 and (**D**,**F**) His2 virus infection (MOI of 20). For PBC^−^ binding and oxygen consumption for the uninfected and heat disrupted control cells, see Figure 2A–C.

We monitored infected and uninfected *Har. hispanica* cell cultures by measuring the culture turbidity, as well as the number of both progeny viruses and viable cells. At 25 h p.i., the uninfected culture reached a turbidity of ~1.4 (A_550_) and a viable cell count of ~2 × 10^9^ cfu/mL (Figure 2A,D and Figure 3A,D).

Following SH1 infection, the decrease in culture turbidity and virus release both started at 6 h p.i. (Figure 2A). At 25 h p.i., the turbidity was ~0.6 (A_550_), the viable cell count was reduced to ~1/1000, and the number of infectious progeny viruses had reached ~2 × 10^11^ pfu/mL (Figure 2A). The lifecycle of HHTV-1 proceeded similarly, except that virus production increased later, at 7 h p.i. and the final virus titer was ~7.5 × 10^10^ pfu/mL (Figure 2D).

In the case of His1, the turbidities of the infected and uninfected *Har. hispanica* cultures did not show any significant differences, and at 25 h p.i., the number of viable cells in both cultures were practically the same (~7 × 10^8^ and ~2 × 10^9^ cfu/mL). Exit of the progeny His1 viruses started 3 to 4 h p.i., and the number of infectious viruses reached ~3 × 10^10^ pfu/mL at 25 h p.i. (Figure 3A).

The progeny His2 viruses appeared 3 to 4 h p.i. and caused cell growth retardation (Figure 3D). At 25 h p.i., the number of infectious progeny viruses was high (~5 × 10^11^ pfu/mL). At the same time, the difference in the numbers of viable cells in the infected and uninfected cultures was around two orders of magnitude (~2 × 10^9^ *vs.* ~3 × 10^7^ cfu/mL; Figure 3D). Based on culture turbidity data (and see also below), neither His1 nor His2 caused any detectable cell lysis during virus release, whereas SH1 and HHTV-1 clearly caused lysis of *Har. hispanica* cells.

### 3.3. Binding of PCB^−^ to *Har. hispanica* Cells as an Indicator of the Start of Lytic Virus Release

The PCB^−^ anion can be effectively used as an indicator of the integrity of the cell envelope as it binds to exposed phospholipid bilayers [48,57,58]. To measure the binding of PCB^−^, the uninfected and infected cells were grown in MGM medium (~3 M NaCl) supplemented with PCB^−^ (3 µM). When the cells were added to the vessels to start the measurements, decrease of PCB^−^ concentration in the medium was observed as a consequence of unspecific binding of this indicator by the culture components defining the background level (Figure 2B). The presence of PCB^−^ in the growth medium decreased the *Har. hispanica* growth rate only slightly during the exponential growth phase and had no effect on progeny production for all four viruses. Both electrodes (see Materials and Methods) gave the same reproducible signal. Maximum binding of PCB^−^ occurred with heat disrupted cells, a treatment that reduced the number of viable cells ~3.5 orders of magnitude (Figure 2B). When monitoring binding during the infection cycle, once equilibrium had been reached, the cells did not bind further PCB^−^ (Figure 2B,E and Figure 3B,E).

During SH1 infection, a change in PCB^−^ binding started at 6 h p.i., the same time as the start of virus release (Figure 2A,B). Similar strong binding of PCB^−^ was observed during HHTV-1 infection but starting at 7 h p.i., which also was simultaneous with the initial virus release (Figure 2D,E). However, during His1 and His2 virus production and release, minimal increase in PCB^−^ binding was observed in spite of the high number of viruses released (Figure 3A,B,D,E).

### 3.4. Changes in the Levels of Oxygen Consumption during Virus Infection

For aerobic organisms, the level of oxygen consumption is a universal measure of the physiological state of the cell. This parameter was assessed by measuring the amount of oxygen dissolved in the medium of infected and uninfected cell cultures. The defined aeration rate used provided sufficient oxygen to sustain the exponential growth rate while also maintaining the oxygen level within a quantifiable range. When the cells were added to the reaction vessel (Figure 2C, arrow), consumption of oxygen started immediately and the amount of dissolved oxygen in the medium decreased rapidly until it reached the balance. As uninfected *Har. hispanica* cells entered stationary phase (~10 h p.i.), their oxygen consumption declined, resulting in first a steady and then an increasing level of oxygen in the medium (Figure 2C). During SH1 infection, oxygen consumption by *Har. hispanica* cells peaked at 6 h p.i., simultaneous with increase of the progeny SH1 virus particles (Figure 2C). After that the registered oxygen level in the medium increased significantly indicating cell death. Similarly, oxygen consumption by the HHTV-1 infected culture decreased starting at 7 h p.i., concurrent with the peak virus production (Figure 2F). In contrast, the production of His1 viruses did not affect oxygen consumption by the host cells (Figure 3C). In the case of His2, a comparable trend was observed, *i.e.*, no change in the oxygen level was evident during virus release, but the infected cells consumed less oxygen than the uninfected ones (Figure 3F). This correlates with the growth rate retardation and the resulting reduced number of viable cells in His2 infected cultures (Figure 3D,F).

### 3.5. Changes in the Concentration of Intracellular and Extracellular ATP during Virus Infection

**Figure 4:**
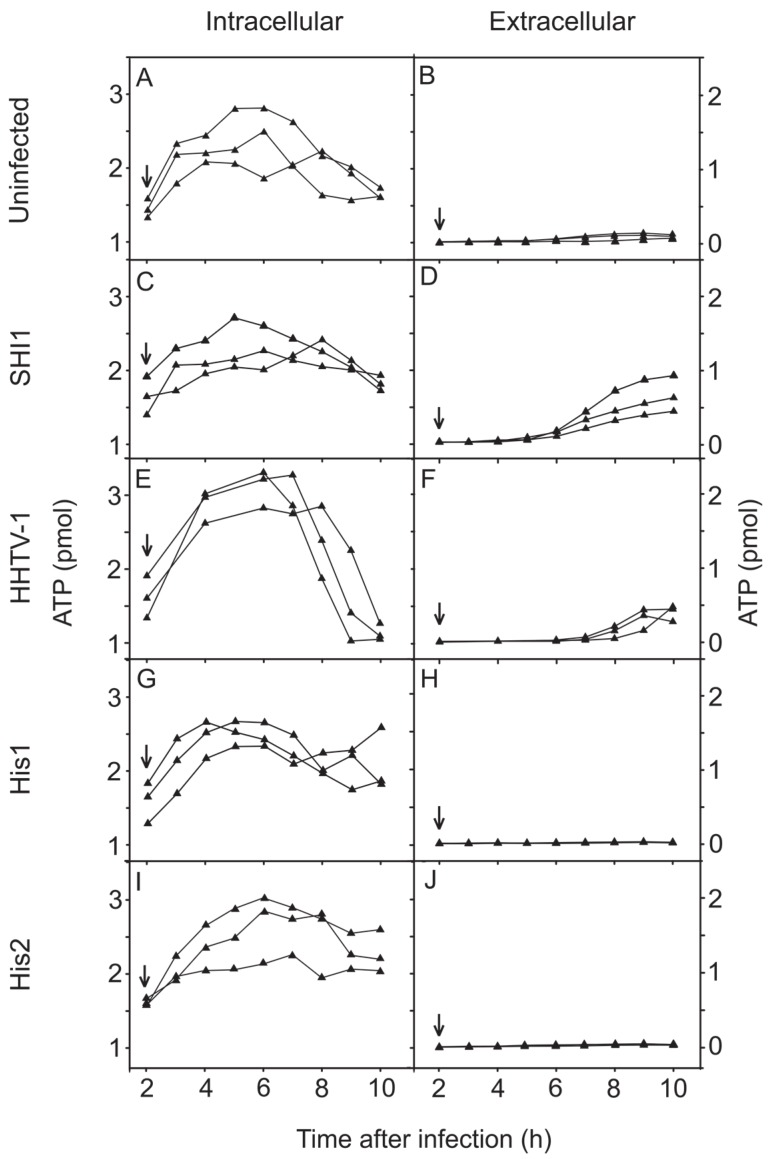
The amount of the intracellular and extracellular ATP in uninfected and virus-infected (MOI of 20) cultures. Unadsorbed viruses were removed at 2 h p.i. and measurements (*n* = 3) were started at 2 h 15 min p.i. (marked by arrows).

ATP content of *Har. hispanica* cells and ATP leakage from the cells into the medium were measured during the first 10 h following infection with each of the four haloarchaeal viruses (Figure 4). For uninfected *Har. hispanica* cells, ATP content was growth phase dependent, decreasing in the late logarithmic phase, and ATP leakage was also observed (Figure 4A,B). Both SH1 and HHTV-1 caused strong leakage of ATP at the time of virus production (Figure 4C–F). Following infection with His1 or His2 (Figure 4G–J), the ATP levels resembled those for uninfected *Har. hispanica* cells (Figure 4B) with no indication of ATP leakage to the extracellular medium.

## 4. Discussion

The first archaeal virus was discovered in 1974 [4], although *Archaea* was not recognized as a separate domain until the late 1970s [60]. Since then, the uniqueness of archaeal viruses and their ability to flourish in extreme environments has inspired researchers to also investigate their interplay with their host cells. The recent isolation of more archaeal viruses [5,6] has offered the possibility of new archaeal virus-host model systems. Nevertheless, the limited set—or complete lack—of genetic tools to manipulate archaeal viruses, e.g., suppression [61,62,63], and the extreme conditions required for their propagation pose significant challenges. Consequently, our knowledge of archaeal virus life cycles is nearly non-existent.

It has been thought for phages that their comparatively early and synchronous lysis reflects their rapid adsorption [64]. Even in high salinity environments, the phages have higher adsorption rates than the haloarchaeal viruses present [29,65]. Pleomorphic virus His2 was found in this study to bind slowly to *Har. hispanica* cells with an adsorption rate constant of 5.0 × 10^−12^ mL/min (Figure 1). This rate is comparable to those of the other three *Har. hispanica* viruses included in this study: icosahedral membrane-containing virus SH1, spindle-shaped virus His1, and icosahedral tailed virus HHTV-1 (1.1 × 10^−11^ to 2.9 × 10^−13^ mL/min; Table 1) [24,29,30]. However, it is considerably slower than the rate for the pleomorphic virus HHPV-1 of *Har. hispanica* (2.0 × 10^−10^ mL/min [29]). By having an adsorption rate constant comparable to that of phages, HHPV-1 demonstrates that not all halophilic archaeal viruses are slow binders [29].

Haloarchaeal virulent viruses are often released slowly and nonsynchronously [31,32,66]. Due to the differences between the cell envelopes of archaea and bacteria [3], the lysis mechanisms of archaeal viruses most probably do not mimic those known for phages [67,68]. Indeed, the pyramid structures induced in the archaeal cell envelopes have been observed only during egress of the crenarchaeal viruses STIV and SIRV2 [9,10]. However, so far no other mechanism for virus-induced disruption of archaeal membranes has been proposed. The slow adsorption rate of these viruses can explain their non-synchronous release by cell lysis. For slowly binding viruses such as SH1 (Table 1), their virus release can be easily misinterpreted as occurring without lysis [9,10]. Consequently, measurement of the infected culture turbidity alone cannot always detect the lytic exit of archaeal viruses. This is also the case for archaeal viruses that use the pyramid exit mechanism due to the creation of empty ghost cells [9,10]. To avoid this pitfall, we took advantage of electrochemical techniques to probe the changes in the integrity of the euryarchaeal cell membrane during virus production and egress and to detect the changes in oxygen consumption. This work represents the first application of this approach to the haloarchaea and their viruses that thrive in high salinity (over 3 M NaCl). We used previously prepared PCB^−^ electrodes, but also developed a new, convenient method to construct PCB^−^ selective electrodes (see Material and Methods). Thus, by observing the impact of archaeal virus infections on the physiological state of *Har. hispanica* cells during virus progeny production, we could determine unambiguously whether or not the virus exit was accompanied by membrane disruption (Figure 2 and Figure 3).

Here we demonstrated that the SH1 virus life cycle ends with the lysis of the *Har. hispanica* host cells and the resultant release of progeny viruses (Figure 2A–C), as had been previously shown [31,47] although other exit strategies had also been proposed [46]. Cell lysis was indicated not only by the drop in the culture turbidity concomitant with progeny virus release, but also by the strong increased binding by PCB^−^ and reduction in oxygen consumption. Moreover, ATP leakage from the cells is in line with these metrics (Figure 4C,D). In the same way, HHTV-1 caused cell lysis during its exit (3 h; Figure 2D,F). During cell lysis, less ATP leaked into the medium than was depleted inside the cells (Figure 4E–F). This led us to hypothesize that part of intracellular ATP was used for other processes related to virus assembly, for example virus genome packaging that requires energy in the form of ATP [69].

Comparison of SH1 and HHTV-1 virus release to that of His1 and His2 highlighted a clear difference in their mechanisms of virus exit. Instead of cell lysis, His1 and His2 progeny viruses were released without apparent cell membrane damage. Previously, it has been shown that His1 does not lyse cells, but can also retard cell growth during virus egress [24,28]. Throughout the infection, oxygen consumption stayed at the same level as in the uninfected culture. The host membrane was inaccessible to the PCB^−^ indicator ion showing that the cell envelope was intact (Figure 3E). In addition, the lack of detectable ATP leakage supports the conclusion that His1 is a non-lytic virus. We hypothesize that His1 buds out from the euryarchaeal cell. During His2 release, the turbidity of the infected culture did not decrease, but host growth was retarded in the late logarithmic growth phase (Figure 3D), as had been previously noticed [23,28]. In addition, during His2 infection, cells used less oxygen than other infected ones (Figure 2C,F and Figure 3C,F) suggesting that that His2 infected cells are more compromised than the other cells. Consequently, the number of viable cells that could make a colony on a plate is lower than expected based on turbidity of the culture. We also observed weak PCB^−^ binding at about 12 h p.i., during the late logarithmic growth phase, but we do not attribute this to virus exit induced cell membrane rupture for two reasons: first, at that time the major virus exit was almost finished; second, no ATP leakage was detected during the first 10 h of infection. The number of His2 viruses released into the medium is high (over 1 × 10^11^ pfu/mL at 10 h p.i.). That the presence of these abundant membrane-containing virions does not induce PCB^−^ binding also shows that the intact virus membranes in the infectious virions are inaccessible to the indicator ion and consequently the PCB^−^ binding registered is a consequence of ruptured cell membranes (Figure 3E). These results demonstrate that viruses His1 and His2 are not released by cell lysis. We envision that these methods can be applied to other archaeal virus systems as well, and thereby help clarify the mechanisms of archaeal virus release from their host cells.

## 5. Conclusions

The monitoring of membrane-associated parameters enabled us to clearly distinguish virus egress by cell lysis from exit without disruption of the cellular membrane. Our results indicated that SH1 and HHTV-1 lysed their host cell, whereas the release mechanism of His1 and His2 is non-lytic.

## Figures and Tables

**Table 1:** *Haloarcula hispanica* SH1, HHTV-1, His1, and His2 viruses used in the study: virion and virus life cycle properties.

Virus	Virion Morphotype	Lipids ^1^	Genome ^3^ (GenBank Acc. No.)	Virus Family ^4^	Adsorption Rate Constant (mL/min)	Virus Release Starts (h p.i.)	Virus Exit	Progeny Viruses at 25 h p.i. (pfu/mL)	References
SH1	Icosahedral	Internal membrane	30,889 bp (AY950802)	*Spherolipoviridae*	1.1 × 10^−11^	~6	Lysis	1.5 × 10^11^	[27,30,31] This study
HHTV-1	Icosahedral tailed	No lipids	49,107 bp (KC292025)	Unclassified siphovirus	2.9 × 10^−13^	~7	Lysis	7 × 10^10^	[29,38] This study
His1	Spindle-shaped	Lipid modified MCP ^2^	14,462 bp (AF191796)	*Salterprovirus* genus ^5^	1.9 × 10^−12^	~4	No lysis	3 × 10^10^	[24,28] This study
His2	Pleomorphic	Membrane envelope	16,067 bp (AF191797)	“Pleolipoviridae”	5.0 × 10^−12^	~4	No lysis	5 × 10^11^	[23,28] This study

^1^ Membrane or lipids in the virion; ^2^ MCP, major capsid protein; ^3^ Linear dsDNA molecules; ^4^ International Committee on Taxonomy of Viruses (ICTV); ^5^ Unassigned family.
